# Supplementary material for: Toolbox of FRET-based c-di-GMP biosensors and its FRET-To-Sort application for genome-wide mapping of c-di-GMP regulation
Source: Nat Commun. 2026 Mar 26;17:2955. doi: 10.1038/s41467-026-71105-8 (PMC13031359; doi:10.1038/s41467-026-71105-8)
Supplement: Supplementary file 1 — Supplementary Information [file 41467_2026_71105_MOESM1_ESM.pdf]

Supplementary Information for

Toolbox of FRET-based c-di-GMP biosensors and its FRET-To-Sort application for genome-wide mapping of c-di-GMP regulation

Liyun Wang, Gabriele Malengo[1], Ananda Sanches-Medeiros[1], Xuanlin Chen[1], Julian Pietsch[1], Nataliya Teteneva[1], Silvia González Sierra[1], Ming C. Hammond[2,3] and Victor Sourjik[1,*]

1. Max Planck Institute for Terrestrial Microbiology & Center for Synthetic Microbiology (SYNMIKRO), Karl-von-Frisch-Straße 14, 35043 Marburg, Germany

2. Department of Chemistry and Henry Eyring Center for Cell & Genome Science, University of Utah, Salt Lake City, Utah 84112, United States

3. Department of Chemistry, University of California, Berkeley, California 94720, United States

[*]Corresponding Author: Victor Sourjik, e-mail: victor.sourjik@mpi-marburg.mpg.de

This PDF file contains:

Supplementary Tables 1-4

Supplementary Figures 1-16

Supplementary References

Supplementary Table 1. Hill coefficient for c-di-GMP binding to biosensors in the toolbox

	Biosensor	Hill coefficient[a] - permeabilized cells	Hill coefficient[b] - purified protein
**High affinity**	D3 (*Syntrophothermus lipocalidus*)	1.6 ± 0.4	2.0 ± 0.4
	F1 (*Thermobrachium celere*)	1.7 ± 0.1	1.4 ± 0.4
	H2 (*Thermoanaerobacterales*)	1.9 ± 0.3	
**Medium Affinity**	A9 (*Massilia sp.*)	1.1 ± 0.0	
	D4 (*Pelotomaculum thermopropionicum*)	1.2 ± 0.3	
	C3 (*Halothermothrix orenii*)	1.1 ± 0.0	1.9 ± 0.5
	A3 (*Thermincola potens*)	1.7 ± 1.1	1.5 ± 0.2
	E1 (*Symbiobacterium thermophilum*)	1.2 ± 0.1	
**Low affinity**	H1 (*Escherichia coli*)	1.3 ± 0.3	
	E8 (*Methylotenera mobilis*)	1.2 ± 0.1	
	F4 (*Thermacetogenium phaeum*)	0.9 ± 0.1	
	B1 (*Pseudomonas putida* strain)	2.3 ± 0.1	
	C1 (*Salmonella typhimurium*)	1.2 ± 0.4	1.4 ± 0.1
	F2 (*Bacillus sporothermodurans*)	1.7 ± 0.4	
	F8 (*Pseudomonas putida*)	1.3 ± 0.2	
	E2 (*Natranaerobius thermophilus*)	1.2 ± 0.2	

[a]Data are presented as mean ± SD. *n* = 3, 4 or 6 biological replicates. [b]Data are presented as mean ± SD. *n* = 3 or 4 biological replicates. [a,b]Source data are provided as a Source Data file.

Supplementary Table 2. Additional mutations with elevated c-di-GMP levels identified by Sanger sequencing

Mutations selected for elevated c-di-GMP levels[a]	
**Insertion location[b]**	**Gene product description**
argE	acetylornithine deacetylase
fliE	flagellar protein
glcB	malate synthase G
gspA	Type II secretion system protein
hicB	antitoxin of the HicA-HicB toxin-antitoxin system / DNA-binding transcriptional repressor
tdcD	propionate kinase
yjhR_nanS	*yjhR*: KpLE2 phage-like element; PLD-like domain-containing protein<br>*nanS*: N-acetyl-9-O-acetylneuraminate esterase
yjhV	putative uncharacterized protein
yncI	putative transposase
yqjC	DUF1090 domain-containing protein

[a]Mutants genotyped by both Sanger sequencing and NGS are shown in Table 2 and Supplementary Data 2.
[b]Underline indicates that the insertion is located between two neighbor genes. All gene names are italicized by convention.

Supplementary Table 3. Additional mutations with reduced c-di-GMP levels identified by Sanger sequencing

**Mutations selected for reduced c-di-GMP levels**[a]	
**Insertion location**[b]	**Gene product description**[b]
clsA_*kch*	***clsA***: cardiolipin synthase A<br>*kch*: voltage-gated K^+ channel
glpQ	glycerophosphoryl diester phosphodiesterase
pinH_*ypjB*	***pinH***: putative invertase fragment<br>*ypiB*: DUF5508 domain-containing protein
purE	N^5-carboxyaminoimidazole ribonucleotide mutase
recJ	ssDNA-specific exonuclease
rhsB	rhs element protein
uspC promotor	universal stress protein C
yaaI	DUF2541 domain-containing protein
yaiZ	DUF2754 domain-containing protein
ybaE	uncharacterized protein YbaE
ybhJ	putative hydratase
yegH	inner membrane protein
yfbP	uncharacterized protein

[a]Mutants genotyped by both Sanger sequencing and NGS are shown in Table 3 and Supplementary Data 3

[b]Underline indicates that the insertion is located between two neighbor genes. Genes labeled in bold indicate that the insertions are located upstream of them. All gene names are italicized by convention.

Supplementary Table 4. Strains and the plasmids used in this study

Strains	Relevant genotype[a]	Reference
W3110	wildtype, RpoS^{+}	[1]
VS701	W3110 Δ*pdeH*	[2]
VS679	W3110 Δ*dgcE*	[2]
VS741	W3110 Δ*pdeH*Δ*dgcE*	[3]
BL21(DE3)	Protein expression strain	[4]
LYW7	W3110 Δ*motA*	This work
LYW15	W3110 Δ*fliC*	This work
LYW65	W3110 Δ*flgM*	This work
LYW66	W3110 Δ*flgM*Δ*motA*	This work
LYW68	W3110 Δ*flgM*Δ*fliC*	This work
LYW79	W3110 Δ*fliG*	This work
LYW81	W3110 Δ*fliH*	This work
LYW85	W3110 Δ*flgM*Δ*fliG*	This work
LYW87	W3110 Δ*flgM*Δ*fliH*	This work
LYW102	W3110 Δ*pdeH*Δ*motA*	This work
LYW104	W3110 Δ*pdeH*Δ*fliC*	This work
LYW106	W3110 Δ*pdeH*Δ*dgcE*Δ*motA*	This work
LYW108	W3110 Δ*pdeH*Δ*dgcE*Δ*fliC*	This work
LYW219	W3110 $dgcE^{GGAAF}$	[5]
LYW220	W3110 Δ*pdeH* $dgcE^{GGAAF}$	This work
LYW221	W3110 Δ*pdeH* $dgcE^{GGAAF}$Δ*motA*	This work
LYW222	W3110 Δ*pdeH* $dgcE^{GGAAF}$Δ*fliC*	This work
**Plasmids**		
pTrc99A	Expression vector; IPTG inducible; Amp^R	[6]
pBAD33	Expression vector; arabinose inducible; Cm^R	[7]
pET28	Expression vector; IPTG inducible; Km^R	[4]
pSIJ8	Provides arabinose inducible λ Red recombineering genes and rhamnose-inducible flippase recombinase required for Km^R removal; Amp^R	[8]
pVS1621	*csgA* promoter controlling GFP expression; Km^R	[9]
pKD45	Encodes kanamycin resistance and the *ccdB* toxin under a rhamnose inducible *rha* promoter; Km^R	[5]
pKD46	Encodes λ Red recombinase; Amp^R	[10]

[a]Amp^R, Cm^R and Km^R indicate ampicillin, chloramphenicol and kanamycin resistance, respectively.

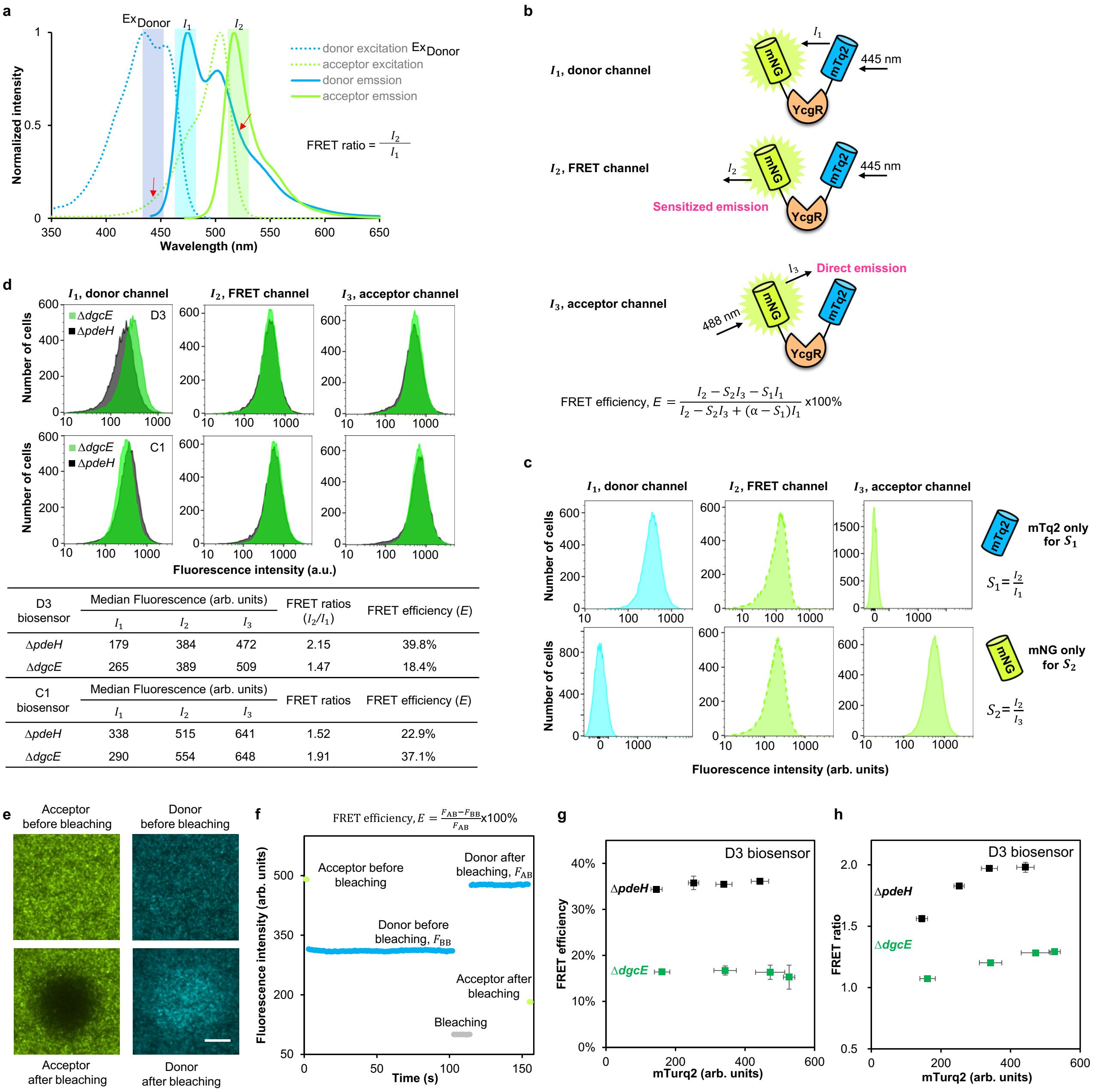

$$\text{FRET efficiency, } E = \frac{I_2 - S_2 I_3 - S_1 I_1}{I_2 - S_2 I_3 + (\alpha - S_1) I_1} \times 100\%$$

$$\text{FRET efficiency, } E = \frac{F_{AB} - F_{BB}}{F_{AB}} \times 100\%$$

D3 biosensor	Median Fluorescence (arb. units) I_1	I_2	I_3	FRET ratios (I_2/I_1)	FRET efficiency (E)
Δ*pdeH*	179	384	472	2.15	39.8%
Δ*dgcE*	265	389	509	1.47	18.4%
**C1 biosensor**	**Median Fluorescence (arb. units) I_1**	**I_2**	**I_3**	**FRET ratios**	**FRET efficiency (E)**
Δ*pdeH*	338	515	641	1.52	22.9%
Δ*dgcE*	290	554	648	1.91	37.1%

Supplementary Fig. 1 Illustration of FRET measurements using YcgR based biosensors. a Excitation and emission spectra of the donor, mTurquoise2 (mTq2), and acceptor, mNeonGreen (mNG), fluorophores, with fluorescence bandpass channels used to excite mTq2 and measure donor (I_1) and acceptor (I_2) emission being indicated. The FRET ratio is defined as the ratio between I_1 and I_2 upon donor excitation. The red arrows respectively indicate the bleed-through into FRET signal from the donor emission (S_1; right) and from the direct excitation of the acceptor (S_2; left). **b** Calculation of the FRET efficiency using flow cytometry data. Flow cytometry is used to measure I_1 and I_2 upon donor excitation, as well as acceptor emission upon acceptor excitation (I_3), and these values are used to calculate the FRET efficiency (E) as shown by the equation. **c** The mTq2-only and mNG-only samples were used to determine the bleed-through parameters S_1 and S_2, respectively. The fluorescence intensity is presented in arbitrary (arb.) units. **d** Histograms of I_1, I_2, I_3 fluorescence intensity for populations of Δ*pdeH* or Δ*dgcE* cells expressing D3 or C1 biosensors, indicated. The table shows the median fluorescence intensities obtained from the histograms, and the calculated values of FRET ratios and FRET efficiency. **e,f** Quantification of FRET efficiency using acceptor photobleaching fluorescence microscopy. Fluorescence of a monolayer of bacteria on a pad was imaged in donor and acceptor fluorescence channels,

before and after acceptor photobleaching (e). Experiments were repeated three times with similar results. Scale bar: 20 µm. FRET efficiency was calculated from acquired donor fluorescence as illustrated in (f), where F_{AB} is donor fluorescence after acceptor photobleaching and F_{BB} is donor fluorescence before acceptor photobleaching. **g,h** The impact of biosensor expression level on the FRET efficiency (g) and on the FRET ratio (h) measured using flow cytometry. n = 3 biological replicates. Source data for a, f, g and h are provided as a Source Data file.

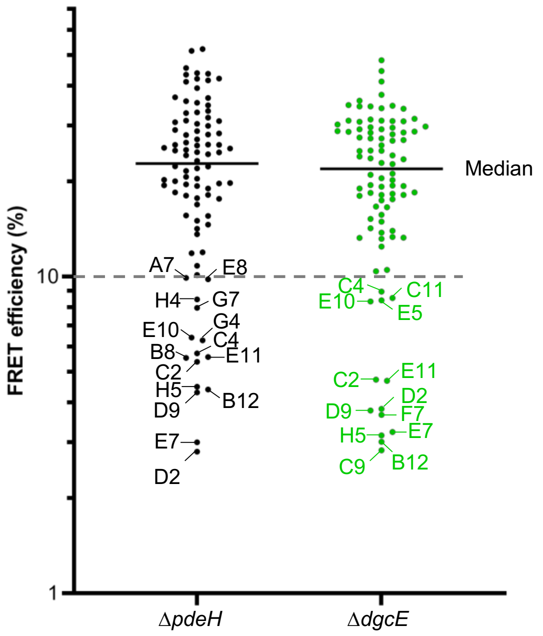

Supplementary Fig. 2 Distributions of FRET efficiency for all biosensors in Δ*pdeH* and in Δ*dgcE* backgrounds. Biosensors with averaged FRET efficiency below 10% are labeled. Source data are provided as a Source Data file.

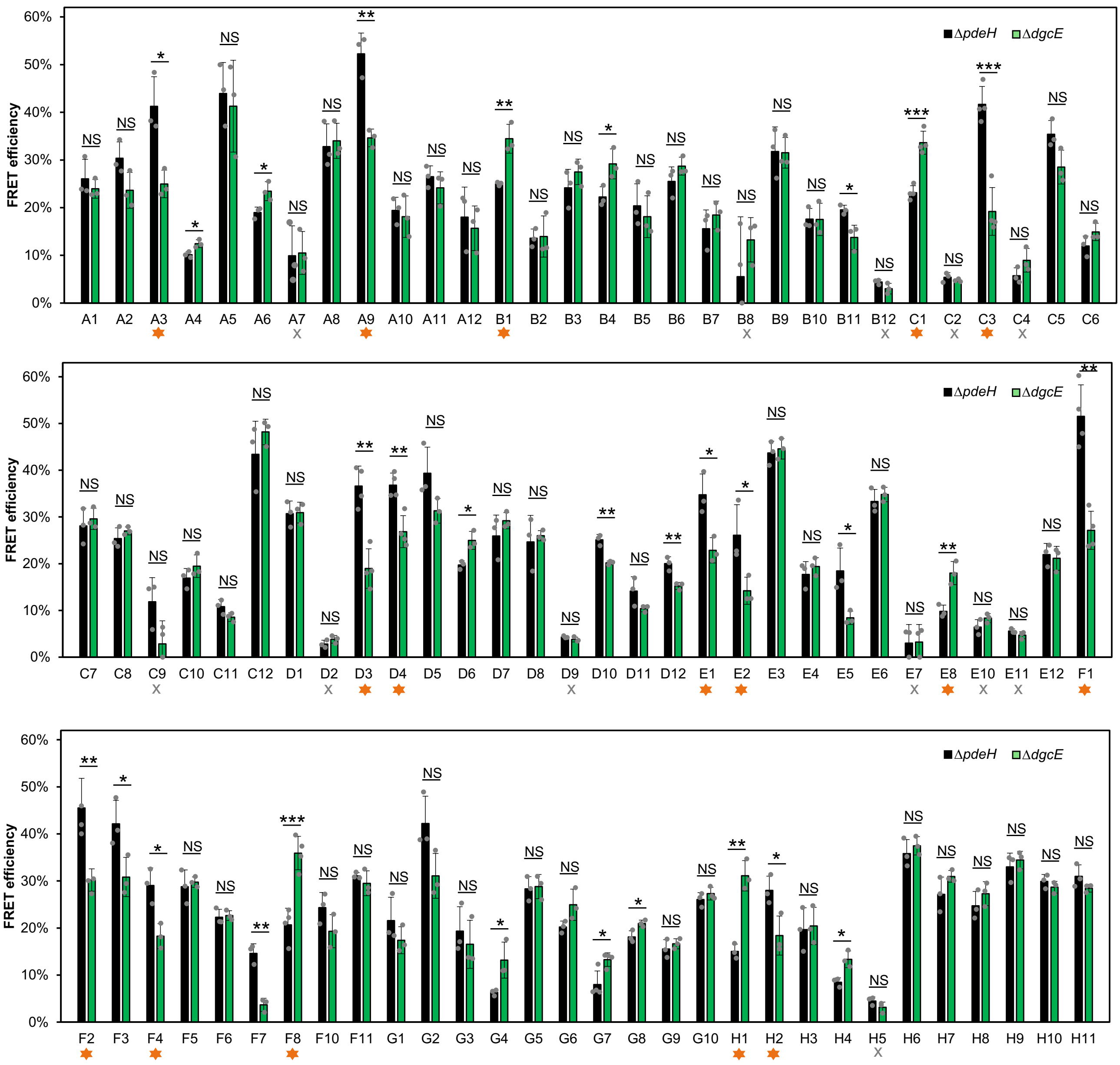

Supplementary Fig. 3 FRET efficiency of each individual biosensor in Δ*pdeH* and in Δ*dgcE* backgrounds. FRET efficiency was determined using flow cytometry. *P* values were calculated using unpaired two-tailed *t-test*, *n* = 3 or 4 biological replicates. * *P* < 0.05; ***P* < 0.01; ****P* < 0.001. Data are presented as mean ± SD. The biosensors labelled with a grey cross underneath were excluded from further analysis due to low values of FRET efficiency (< 10%) or high coefficient of variances in both deletion strains. The biosensors selected for the final toolbox are labelled with an orange star. Note that absolute values of FRET efficiency presented here and Figure 1e slightly differ from those in other figures due to readjustments in the flow cytometer subsequent to these measurements. Exact *P* values for all statistical comparisons are reported in the Source Data file. Source data are provided as a Source Data file.

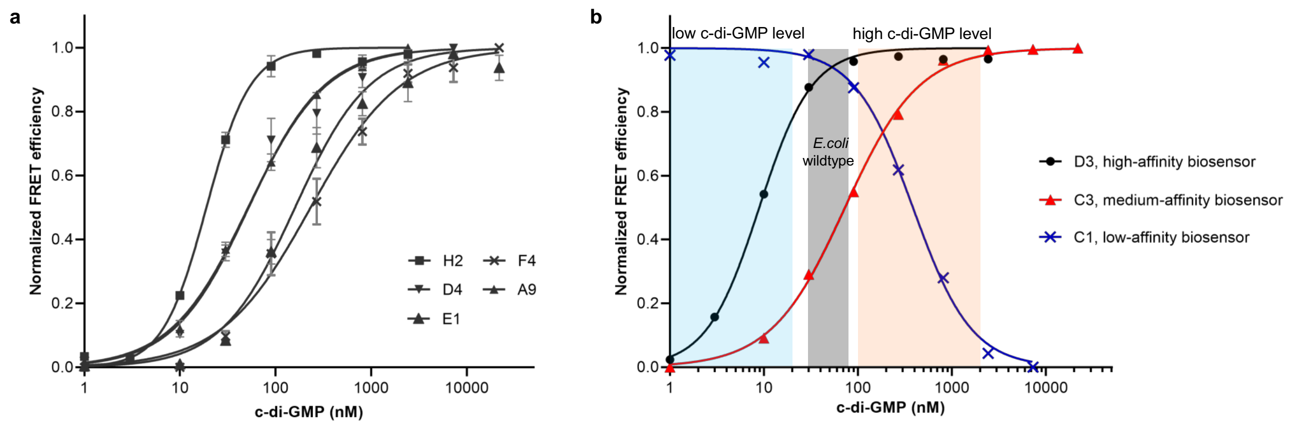

Supplementary Fig. 4 Dose responses of biosensors to c-di-GMP. a Responses of indicated biosensors to c-di-GMP, measured in permeabilized Δ*dgcE* cells. Data are presented as mean ± SEM. *n* = 3 or 4 biological replicates. Only biosensors chosen for the toolbox and not included in Fig. 2b are shown. **b** Illustration of c-di-GMP measurements using high-affinity (black), medium-affinity (red) or low-affinity (blue) biosensors. The range of c-di-GMP levels previously reported in wildtype *E.coli* cells[11] or estimated from biosensor saturation in our *in-vivo* measurements is indicated in grey; the ranges corresponding to low and high c-di-GMP levels, are highlighted respectively in blue and orange. Source data for a is provided as a Source Data file.

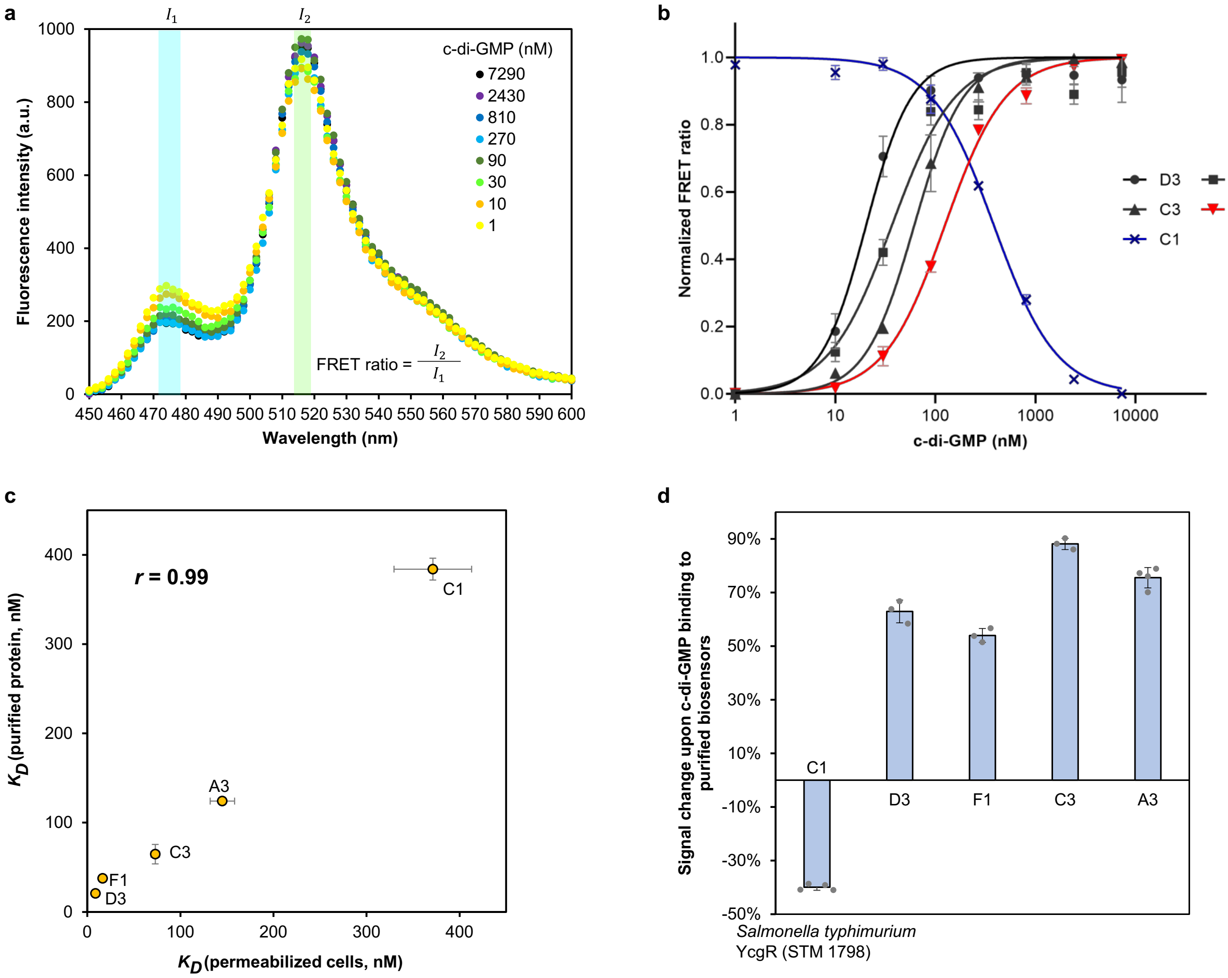

Supplementary Fig. 5 Binding properties of purified biosensor proteins. a Fluorescence emission spectra of purified F1 biosensor at indicated c-di-GMP concentrations. The FRET ratio is calculated as I_2/I_1, where I_2 represents the average fluorescence intensity within the 514-518 nm bandwidth and I_1 represents the average intensity within the 472-478 nm bandwidth. **b** Dose responses of selected biosensors to c-di-GMP, measured using changes of the FRET ratio obtained from the emission spectra. Data are presented as mean ± SEM. n = 3 or 4 biological replicates. **c** Comparison of the K_D values obtained for purified biosensor with those obtained using permeabilized Δ*dgcE* cells. Pearson correlation coefficient r is shown. Data are presented as mean ± SEM. n = 3 or 4 biological replicates for purified protein and n = 3, 4 or 6 biological replicates for permeabilized cells. **d** Maximum signal change upon c-di-GMP binding to the purified biosensor. Data are presented as mean ± SD. n = 3 or 4 biological replicates. Source data for a, b, c and d are provided as a Source Data file.

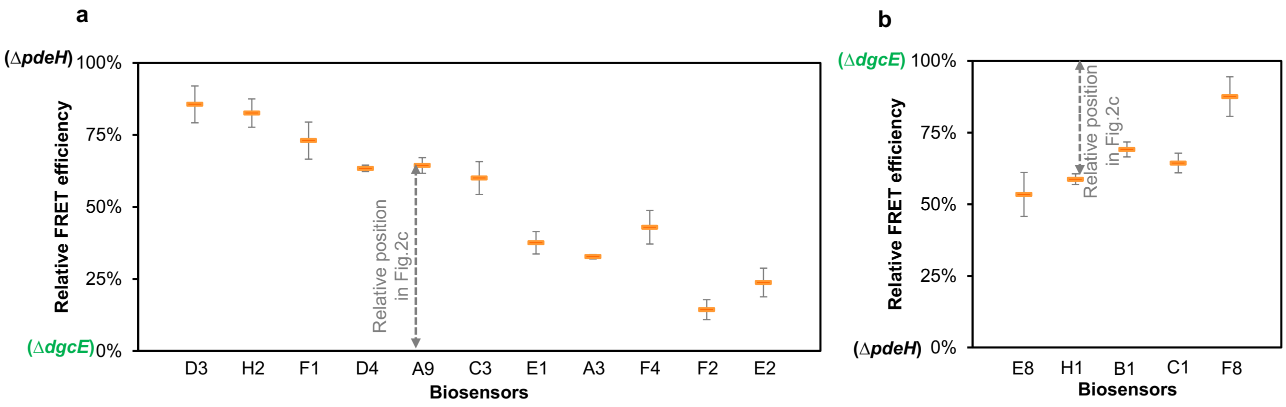

Supplementary Fig. 6 Illustration of the relative sensor occupancy of the wildtype cells. a For biosensors showing increased FRET signals after c-di-GMP binding, the FRET efficiency in Δ*dgcE* and in Δ*pdeH* was set to 0 and 100%, respectively. **b** For biosensors showing opposite conformation change, i.e., lower FRET signals upon c-di-GMP binding, the FRET efficiency in Δ*dgcE* and in Δ*pdeH* was set to 100% and 0, respectively. The relative FRET efficiency in the wildtype is always between 0 and 100%, with the distance between Δ*dgcE* and the wildtype values defined as the relative occupancy, indicated using the grey dash arrow, and displayed in Fig. 2d. Data are presented as mean ± SEM. n = 3 biological replicates. Source data for a and b are provided as a Source Data file.

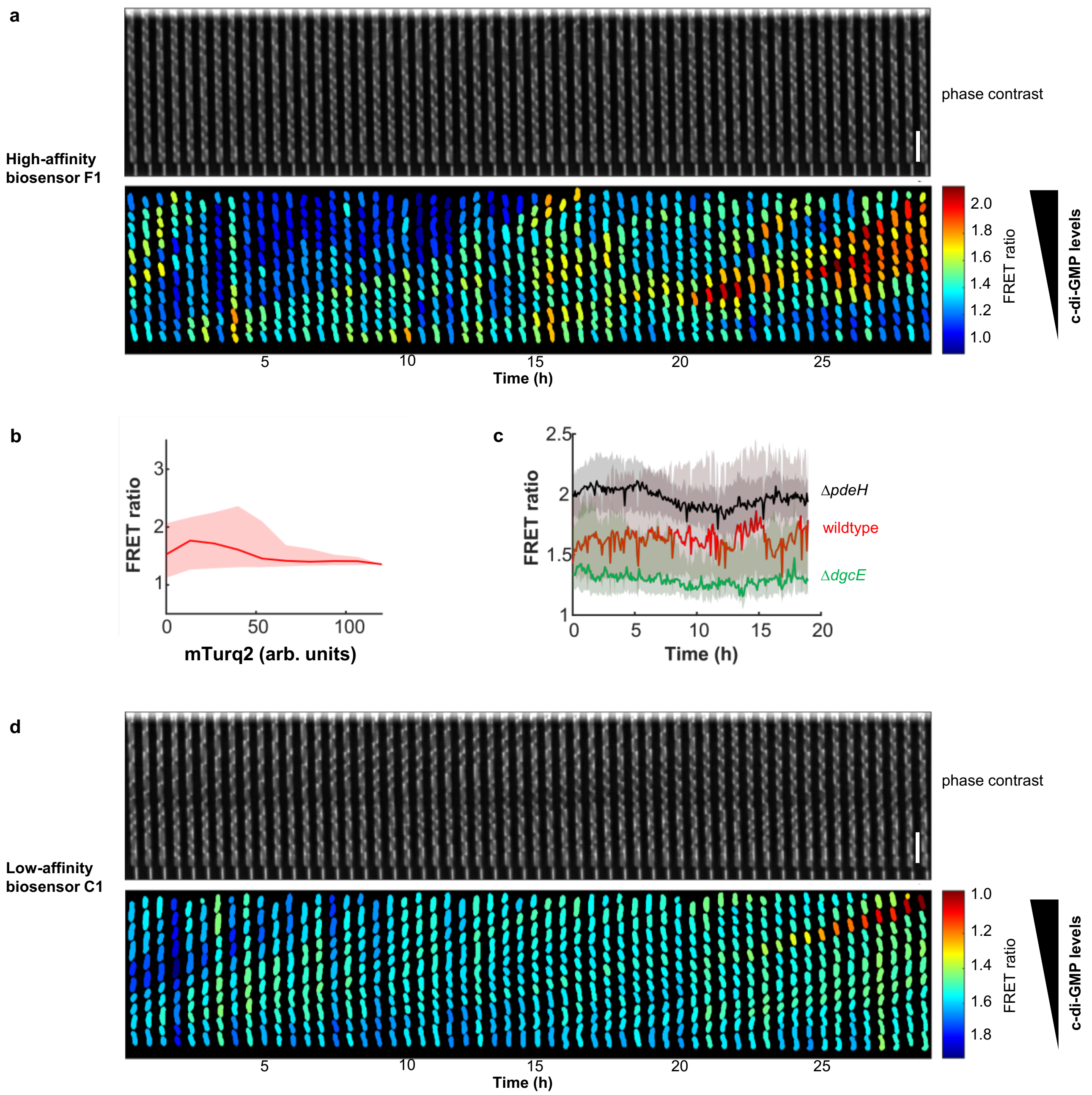

Supplementary Fig. 7 FRET measurements of c-di-GMP levels in *E. coli* cells growing in the mother machine. a Kymograph heat map shows FRET ratio in cells growing in one trench of the mother machine microfluidic device (phase-contrast images above). Measurements made using the high-affinity F1 biosensor. Higher FRET ratio indicates higher c-di-GMP levels. Experiments were repeated twice with similar results. Scale bar: 5 µm. **b** The relation between F1 biosensor expression level and the single-cell FRET ratio in wildtype cells. The shaded area represents the interquartile range, and the solid line indicates the median. **c** FRET ratio values in the populations of Δ*pdeH* cells (black), wildtype cells (red) and Δ*dgcE* cells (green), measured in the same microfluidics experiment and plotted over time. The shaded area represents the interquartile range, and the solid line indicates the median. **d** Kymograph heat map for the microfluidics experiment using low-affinity biosensor C1, which is based on the same YcgR as the previously published FRET biosensor[12,13]. In this case, lower FRET ratio indicates higher c-di-GMP levels. Experiments were repeated twice with similar results. Scale bar: 5 µm. Source data for b and c are provided as a Source Data file.

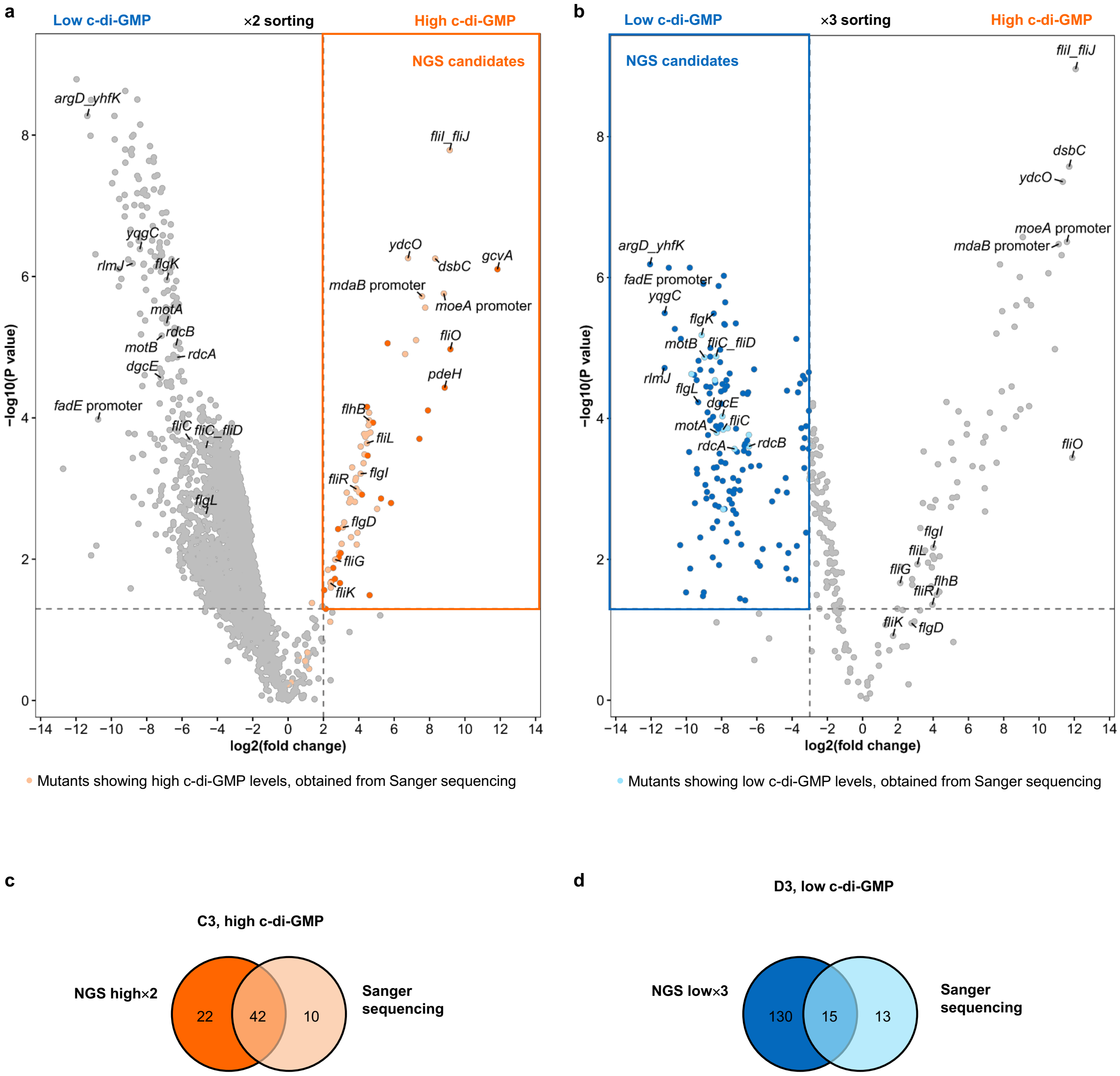

Supplementary Fig. 8 Enriched mutations identified by FRET-to-Sort followed by NGS analysis. a,b Volcano plots of NGS data showing the enrichment difference between sorting for high and low c-di-GMP levels. Data for the second (×2; **a**) and the third (×3; **b**) sorting cycle are shown. $n = 2$ biological replicates. Enriched high c-di-GMP mutations in the ×2 sorting cycle (orange rectangle) and low c-di-GMP mutations in the ×3 sorting cycle (blue rectangle) are highlighted. The enriched mutations are listed in Tables 2 and 3, and in Supplementary Data 2 and 3. Labeled genes are selected candidates shown in Fig. 3d,e and in Tables 2 and 3, and Supplementary Data 2 and 3. Exact P values are reported in the Source Data file. **c,d** Venn Diagrams showing the overlap between mutations identified in Sanger sequencing data and NGS data. Source data for a and b are provided as a Source Data file.

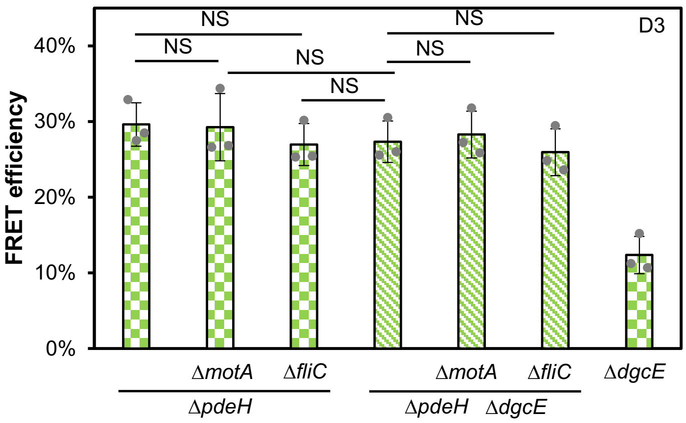

Supplementary Fig. 9 Measurements of c-di-GMP levels in indicated mutants using the high-affinity D3 biosensor. In contrast to the measurements using the lower-affinity C3 biosensor in Fig. 4c, the effects of flagellar class III gene deletions on c-di-GMP in the Δ*pdeH* background could not be reliably resolved, likely due to the D3 biosensor saturation. Higher FRET efficiency indicates higher c-di-GMP levels. *P* values were calculated using unpaired two-tailed *t-test*, $n = 3$ biological replicates. ***P* < 0.01; NS, no significant difference. Data are presented as mean ± SD. Exact *P* values for all statistical comparisons are reported in the Source Data file. Source data are provided as a Source Data file.

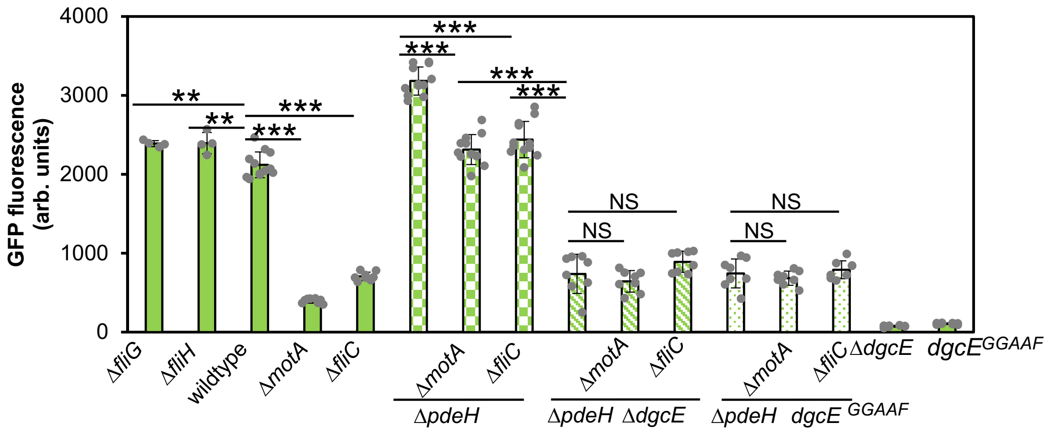

Supplementary Fig. 10 Impact of deletions of flagellar genes on curli expression. Activity of the plasmid-based GFP curli gene expression reporter, transformed in indicated strains, measured using flow cytometry. See main text for the description of individual deletion strains. *P* values were calculated using unpaired two-tailed *t-test*, *n* = 4, 8, or 12 biological replicates. ****P* < 0.001; ***P* < 0.01; **P* < 0.05; NS, no significant difference. Data are presented as mean ± SD. Exact *P* values for all statistical comparisons are reported in the Source Data file. Source data are provided as a Source Data file.

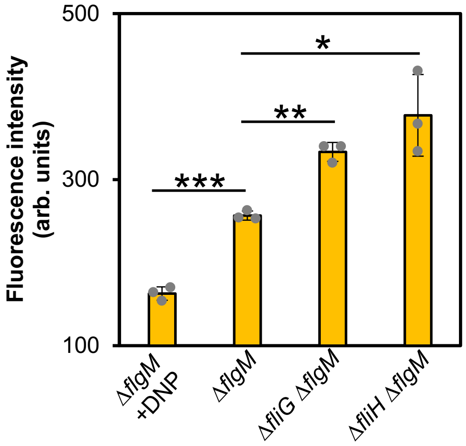

Supplementary Fig. 11 Effect of flagellar class II mutants on the membrane potential. Membrane potential measurements in Δ*flgM* background strains. Membrane potential was measured using potential-sensitive ThT dye, as in Fig. 4d, and is presented in arbitrary (arb.) units. DNP treatment was used as a control. *P* values were calculated using paired two-tailed *t-test*, *n* = 3 biological replicates. ***$P < 0.001$; **$P < 0.01$; *$P < 0.05$; Data are presented as mean ± SD. Exact *P* values for all statistical comparisons are reported in the Source Data file. Source data are provided as a Source Data file.

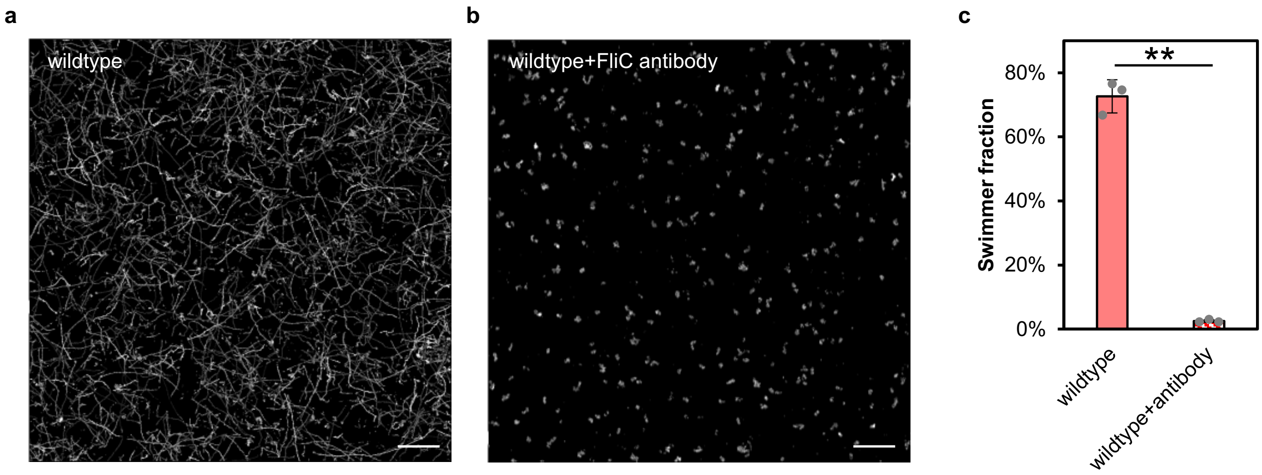

Supplementary Fig. 12 Effect of FliC antibody on swimming motility. a,b Swimming trajectories of wildtype cells without (a) or with (b) FliC antibody treatment. Scale bars, 70 µm. Experiments were repeated twice with similar results. **c** Calculated fraction of swimming cells. *P* values were calculated using paired two-tailed *t-test*, $n = 3$ biological replicates. $^{**}P < 0.01$; Data are presented as mean ± SD. Exact *P* values for all statistical comparisons are reported in the Source Data file. Source data for c is provided as a Source Data file.

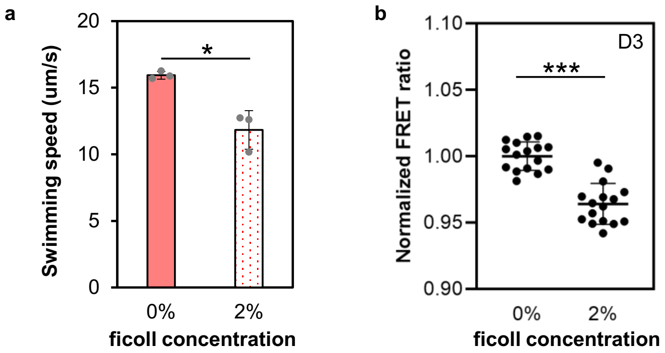

Supplementary Fig. 13 Effect of high media viscosity on swimming motility and c-di-GMP levels. a Swimming speed of wildtype cells without (0%) or with (2%) ficoll in the buffer. *P* values were calculated using paired two-tailed *t-test*, $n = 3$ biological replicates. *$P < 0.05$; Data are presented as mean ± SD. **b** C-di-GMP levels in wildtype cells without (0%) or with (2%) ficoll in the buffer. Higher FRET ratio indicates higher c-di-GMP levels. FRET ratio was normalized by the mean value of FRET ratios in 0% ficoll. *P* values were calculated using unpaired two-tailed *t-test*, $n = 16$ (four biological replicates, each measured with four technical replicates). ***$P < 0.001$; Data are presented as mean ± SD. Exact *P* values for all statistical comparisons are reported in the Source Data file. Source data for a and b are provided as a Source Data file.

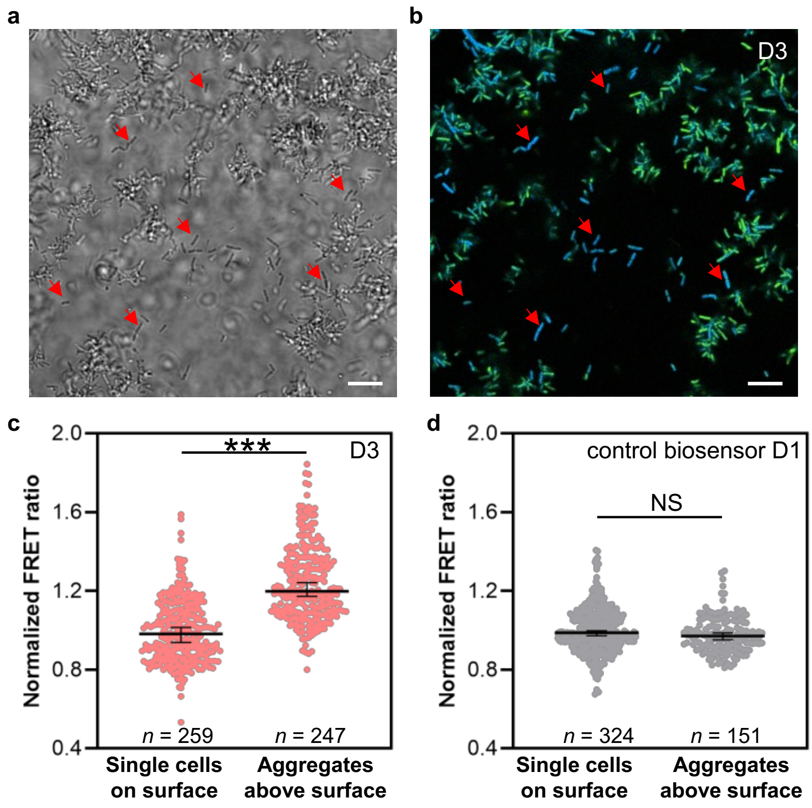

Supplementary Fig. 14 C-di-GMP levels in surface-attached cells and in cell aggregates above the surface. **a** Bright-field image showing single cells attached on the surface and cell aggregates above the surface. The focus plane is above the surface. **b** Lambda-mode image of the same field of view as in (a), with the focal plane on the surface. Cells appearing bluer indicate lower FRET signals, whereas greener cells indicate higher FRET signals. Scale bars, 10 µm. Red arrows indicate representative single cells attached to the surface. For (a) and (b), experiments were repeated three times with similar results. **c,d** C-di-GMP levels in single surface-attached cells and in cells within aggregates above the surface, measured using the D3 biosensor (c) and the unresponsive D1 biosensor (see Supplementary Fig. 3) as a control (d). In (c), higher FRET ratios indicate higher c-di-GMP levels. For each field of view, images were acquired at different z-positions, focusing either on single cells attached to the surface or on cell aggregates above the surface. FRET ratios were normalized to the mean FRET ratio of single surface-attached cells. *P* values were calculated using the Mann-Whitney U test; ***P* < 0.001; NS, not significant. Data are presented as median ± 95% confidence interval. The *n* values represent the number of cells or of aggregates analyzed, and data are from three biological replicates, each with three technical replicates. Exact *P* values for all statistical comparisons are reported in the Source Data file. Source data for c and d are provided as a Source Data file.

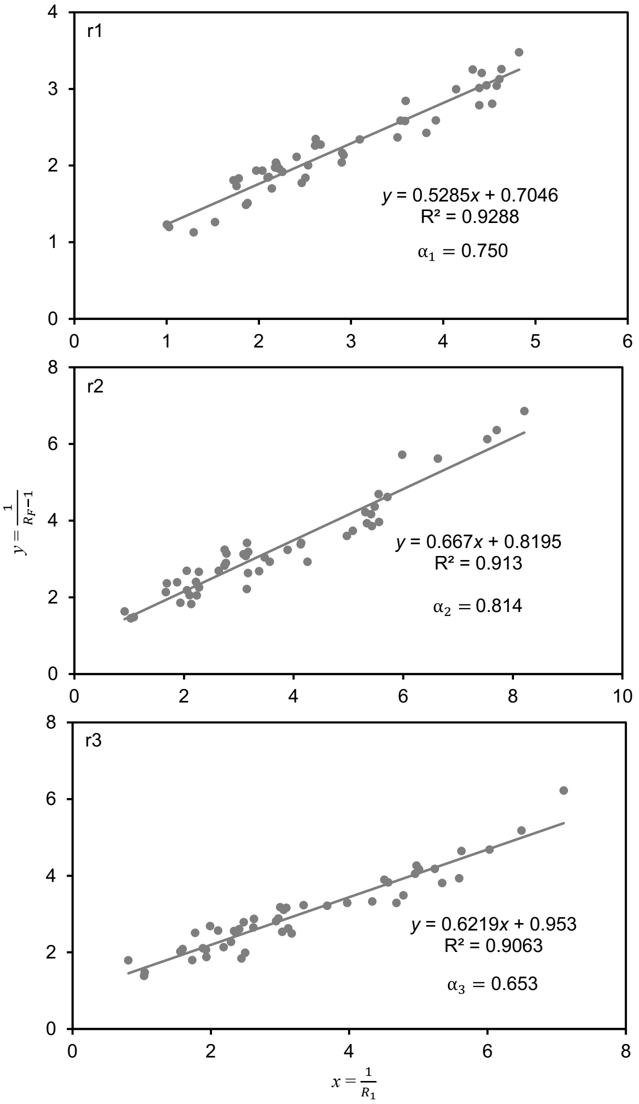

Supplementary Fig. 15 Calibration factor α for the calculation of FRET efficiency determined from flow cytometry FRET data. The linear regressions provide the slope and the intercept from the plot of $y = \frac{1}{R_F - 1}$ vs $x = \frac{1}{R_1}$. See Methods for details. The ratio of the slope and the intercept yields the value of α. The r1, r2 and r3 represent independent biological replicates. Source data are provided as a Source Data file.

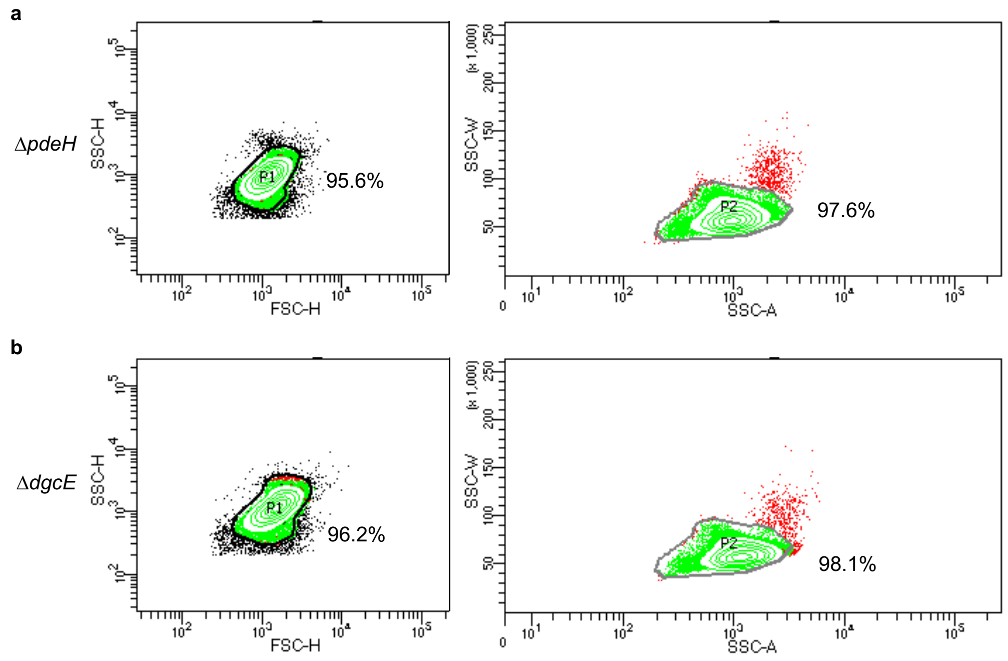

Supplementary Fig. 16 Flow cytometry gating strategy. a,b Forward scatter (FSC) and side scatter (SSC) plots for flow cytometry measurements of Δ*pdeH* (a) and Δ*dgcE* (b) cell populations. Gates P1 and P2 illustrate the gating strategy used for flow cytometry analysis. Percentages represent the fraction of gated cells within population. P2 is the subpopulation of P1. Cells were distinguished by size based on FSC-H versus SSC-H plot, and doublets were excluded using SSC-A versus SSC-W plot.

Supplementary References:

1 Serra, D. O., Richter, A. M., Klauck, G., Mika, F. & Hengge, R. Microanatomy at cellular resolution and spatial order of physiological differentiation in a bacterial biofilm. *MBio* **4**, e00103-00113 (2013).

2 Suchanek, V. M. *et al.* Chemotaxis and cyclic-di-GMP signalling control surface attachment of *Escherichia coli*. *Mol. Microbiol.* **113**, 728-739 (2020).

3 Suchanek, V. M. *Role of Motility and its Regulation in Escherichia coli Biofilm formation*, (2017).

4 Xu, W. *et al.* Systematic mapping of chemoreceptor specificities for *Pseudomonas aeruginosa*. *Mbio* **14**, e02099-02023 (2023).

5 Pfiffer, V., Sarenko, O., Possling, A. & Hengge, R. Genetic dissection of *Escherichia coli*'s master diguanylate cyclase DgcE: Role of the N-terminal MASE1 domain and direct signal input from a GTPase partner system. *PLoS Genet.* **15**, e1008059 (2019).

6 Amann, E., Ochs, B. & Abel, K.-J. Tightly regulated tac promoter vectors useful for the expression of unfused and fused proteins in *Escherichia coli*. *Gene* **69**, 301-315 (1988).

7 Guzman, L.-M., Belin, D., Carson, M. J. & Beckwith, J. Tight regulation, modulation, and high-level expression by vectors containing the arabinose PBAD promoter. *J. Bacteriol.* **177**, 4121-4130 (1995).

8 Jensen, S. I., Lennen, R. M., Herrgård, M. J. & Nielsen, A. T. Seven gene deletions in seven days: Fast generation of *Escherichia coli* strains tolerant to acetate and osmotic stress. *Sci. Rep.* **5**, 17874 (2015).

9 Lamprecht, O. *Heterogeneity of gene expression during biofilm formation in Escherichia coli*, Philipps-Universität Marburg Marburg, (2018).

10 Datsenko, K. A. & Wanner, B. L. One-step inactivation of chromosomal genes in *Escherichia coli* K-12 using PCR products. *Proc. Natl. Acad. Sci. U.S.A.* **97**, 6640-6645 (2000).

11 Sarenko, O. *et al.* More than enzymes that make or break cyclic di-GMP-local signaling in the interactome of GGDEF/EAL domain proteins of *Escherichia coli*. *MBio* **8**, e01639-01617 (2017).

12 Christen, M. *et al.* Asymmetrical distribution of the second messenger c-di-GMP upon bacterial cell division. *Science* **328**, 1295-1297 (2010).

13 Pultz, I. S. *et al.* The response threshold of *Salmonella* PilZ domain proteins is determined by their binding affinities for c-di-GMP. *Mol. Microbiol.* **86**, 1424-1440 (2012).
