# Supplementary material for: Toolbox of FRET-based c-di-GMP biosensors and its FRET-To-Sort application for genome-wide mapping of c-di-GMP regulation
Source: Nat Commun. 2026 Mar 26;17:2955. doi: 10.1038/s41467-026-71105-8 (PMC13031359; doi:10.1038/s41467-026-71105-8)
Supplement: Supplementary file 2 — Description of Additional Supplementary Information [file 41467_2026_71105_MOESM2_ESM.pdf]

Description of Additional Supplementary Files

File Name: Supplementary Data 1
Description: List of YcgR homologues containing proteins used in the biosensor library.

File Name: Supplementary Data 2
Description: Additional mutations with elevated c-di-GMP levels identified by NGS.

File Name: Supplementary Data 3
Description: Additional mutations with reduced c-di-GMP levels identified by NGS.

File Name: Supplementary Data 4
Description: Oligonucleotide primers used for mutagenesis and plasmid construction.

File Name: Supplementary Movie 1
Description: **Time-lapse movie of wildtype E. coli cells swimming in the control buffer.** Images were acquired with an exposure time of 1 ms at a rate of 50 frames per second (fps) for 40 s. Scale bars, 70 µm.

File Name: Supplementary Movie 2
Description: **Time-lapse movie of wildtype E. coli cells with flagellar rotation inhibited by FliC antibody treatment.** Images were acquired with an exposure time of 1 ms at a rate of 50 frames per second (fps) for 40 s. Scale bars, 70 µm.

File Name: Supplementary Movie 3
Description: **Confocal microscopy z-stacks of wildtype E. coli cells at an early stage of surface colonization and aggregate formation**. Step size, 0.7 µm. Scale bars, 10 µm.
