# Supplementary material for: Toolbox of FRET-based c-di-GMP biosensors and its FRET-To-Sort application for genome-wide mapping of c-di-GMP regulation
Source: Nat Commun. 2026 Mar 26;17:2955. doi: 10.1038/s41467-026-71105-8 (PMC13031359; doi:10.1038/s41467-026-71105-8)
Supplement: Supplementary file 10 — Reporting Summary [file 41467_2026_71105_MOESM10_ESM.pdf]

nature portfolio

Corresponding author(s): Victor Sourjik

Last updated by author(s): Mar 10, 2026

Reporting Summary

Nature Portfolio wishes to improve the reproducibility of the work that we publish. This form provides structure for consistency and transparency in reporting. For further information on Nature Portfolio policies, see our Editorial Policies and the Editorial Policy Checklist.

Statistics

For all statistical analyses, confirm that the following items are present in the figure legend, table legend, main text, or Methods section.

n/a	Confirmed	
☐	☒	The exact sample size (*n*) for each experimental group/condition, given as a discrete number and unit of measurement
☐	☒	A statement on whether measurements were taken from distinct samples or whether the same sample was measured repeatedly
☐	☒	The statistical test(s) used AND whether they are one- or two-sided<br>*Only common tests should be described solely by name; describe more complex techniques in the Methods section.*
☒	☐	A description of all covariates tested
☒	☐	A description of any assumptions or corrections, such as tests of normality and adjustment for multiple comparisons
☐	☒	A full description of the statistical parameters including central tendency (e.g. means) or other basic estimates (e.g. regression coefficient) AND variation (e.g. standard deviation) or associated estimates of uncertainty (e.g. confidence intervals)
☐	☒	For null hypothesis testing, the test statistic (e.g. *F*, *t*, *r*) with confidence intervals, effect sizes, degrees of freedom and *P* value noted<br>*Give P values as exact values whenever suitable.*
☐	☒	For Bayesian analysis, information on the choice of priors and Markov chain Monte Carlo settings
☒	☐	For hierarchical and complex designs, identification of the appropriate level for tests and full reporting of outcomes
☐	☒	Estimates of effect sizes (e.g. Cohen's *d*, Pearson's *r*), indicating how they were calculated

Our web collection on statistics for biologists contains articles on many of the points above.

Software and code

Policy information about availability of computer code

Data collection	Photobleaching microscopy data and ratiometric FRET microscopy data were acquired with NIS-Elements AR software (version 4.40, Nikon). Mother machine microscopy data were acquired with NIS-Elements AR software (version 5.20, Nikon). Confocal microscopy data were acquired with Zeiss Zen software (version 2.1 SP3, Zeiss). Microscopy for motility measurement were acquired with StreamPix 6 software (version multi-camera, NorPix). Flow cytometry/FACS data were acquired with BD FACSDiva Software (version v8.0.1, BD). Plate reader data were acquired with i-control software (version 2.0, Tecan)
Data analysis	Statistics were analyzed using SPSS Statistics (Version 29.0.2.0, IBM), Microsoft Excel 2021 (Microsoft), and GraphPad Prism (Version 9.0, Graphpad software Inc.). Statistics of NGS data were analyzed in Cyber-T web server (Kayala, M. A. & Baldi, P. Nucleic Acids Res. 40, W553-W559, 2012).<br>Flow cytometry data were exported using FlowJo (version 10.10.0, BD).<br>Mother machine image analysis was conducted using Matlab R2023a, and the code is available from https://git.ecdf.ed.ac.uk/swain-lab/baby.<br>Images using Lambda Mode in confocal microscopy were exported using Zeiss ZEN lite software (version 3.13, Zeiss).<br>The networks generated using the STRING database were visualized using Cytoscape (version 3.9.1, NHGRI).<br>The fraction of swimming cells was determined by Differential Dynamic Microscopy (DDM) using plugins for ImageJ, and the code is available from https://doi.org/10.5281/zenodo.3516258. The swimming trajectories of cells were analyzed using the Particle_Tracking_2 ImageJ Plugin, and the code is available from https://doi.org/10.5281/zenodo.18890231.

For manuscripts utilizing custom algorithms or software that are central to the research but not yet described in published literature, software must be made available to editors and reviewers. We strongly encourage code deposition in a community repository (e.g. GitHub). See the Nature Portfolio guidelines for submitting code & software for further information.

Data

Policy information about availability of data

All manuscripts must include a data availability statement. This statement should provide the following information, where applicable:

- Accession codes, unique identifiers, or web links for publicly available datasets
- A description of any restrictions on data availability
- For clinical datasets or third party data, please ensure that the statement adheres to our policy

NGS datasets used in this study are available in the NCBI database under accession BioProject PRJNA1144054 [http://www.ncbi.nlm.nih.gov/bioproject/1144054]. Flow cytometry and microscopy raw data are available from [https://doi.org/10.17617/3.BFOX3H]95. The data presented in the paper and the Supplementary Information are available in the Source Data file. Source data are provided with this paper. The biological materials are available upon request under a Material Transfer Agreement.

Research involving human participants, their data, or biological material

Policy information about studies with human participants or human data. See also policy information about sex, gender (identity/presentation), and sexual orientation and race, ethnicity and racism.

Reporting on sex and gender	n/a
Reporting on race, ethnicity, or other socially relevant groupings	n/a
Population characteristics	n/a
Recruitment	n/a
Ethics oversight	n/a

Note that full information on the approval of the study protocol must also be provided in the manuscript.

Field-specific reporting

Please select the one below that is the best fit for your research. If you are not sure, read the appropriate sections before making your selection.

☒ Life sciences ☐ Behavioural & social sciences ☐ Ecological, evolutionary & environmental sciences

For a reference copy of the document with all sections, see nature.com/documents/nr-reporting-summary-flat.pdf

Life sciences study design

All studies must disclose on these points even when the disclosure is negative.

Sample size	No statistical methods were used to predetermine the sample size. Flow cytometry experiments acquired 30,000 events per sample. For FRET-To-Sort sorting, 50,000 cells per sample were collected.
Data exclusions	No
Replication	For statistics analysis, generally, at least three biological replicates were performed, except for the FRET-To-Sort enrichment that was performed in two biological replicates because of the experimental complexity.
Randomization	No. The experimental results were not randomized.
Blinding	Only NGS data were analyzed in a blinding way (FRET-To-Sort was performed by L.W. and the following NGS was analyzed by A.S.M.). Since flow cytometry data acquired using same settings and quantitative analyses were performed using the same gating strategies, blinding was not relevant.

Reporting for specific materials, systems and methods

We require information from authors about some types of materials, experimental systems and methods used in many studies. Here, indicate whether each material, system or method listed is relevant to your study. If you are not sure if a list item applies to your research, read the appropriate section before selecting a response.

Materials & experimental systems

n/a	Involved in the study
[ ]	[x] Antibodies
[x]	[ ] Eukaryotic cell lines
[x]	[ ] Palaeontology and archaeology
[x]	[ ] Animals and other organisms
[x]	[ ] Clinical data
[x]	[ ] Dual use research of concern
[x]	[ ] Plants

Methods

n/a	Involved in the study
[x]	[ ] ChIP-seq
[ ]	[x] Flow cytometry
[x]	[ ] MRI-based neuroimaging

Antibodies

Antibodies used	Anti flagellin rabbit polyclonal FITC conjugate primary antibody (Antikoerper; AA 2-498-FITC, ABIN2831532, 1.5 mg/mL).
Validation	For the primary antibody AA 2-498-FITC, validation was done by the manufacturer (https://www.antikoerper-online.de/antibody/1992301/anti-Flagellin+FliC+AA+2-498+antibody+FITC/).

Plants

Seed stocks	n/a
Novel plant genotypes	n/a
Authentication	n/a

Flow Cytometry

Plots

Confirm that:

- [x] The axis labels state the marker and fluorochrome used (e.g. CD4-FITC).
- [x] The axis scales are clearly visible. Include numbers along axes only for bottom left plot of group (a 'group' is an analysis of identical markers).
- [ ] All plots are contour plots with outliers or pseudocolor plots.
- [x] A numerical value for number of cells or percentage (with statistics) is provided.

Methodology

Sample preparation	Sample preparation was described in Methods.
Instrument	BD LSRFortessa SORP; BD FACSAriaTM Fusion
Software	BD FACSDiva software (version v8.0.1)
Cell population abundance	For biosensor screening using flow cytometry, 30,000 events were acquired per sample. For FRET-To-Sort using FACS, 1-2% of the population was selected, collecting 50,000 cells per sample.
Gating strategy	Gating strategy is illustrated in Supplementary Fig. 16, with gating in SSC-A/SSC-W for doublet exclusion and in FSC-H/SSC-H for distinguishing cells by size.

- [x] Tick this box to confirm that a figure exemplifying the gating strategy is provided in the Supplementary Information.
